# Characteristics and differences of gut microbiota in patients with different Traditional Chinese Medicine Syndromes of Colorectal Cancer and normal population

**DOI:** 10.7150/jca.50318

**Published:** 2020-10-23

**Authors:** Peipei Wang, Shuning Ding, Leitao Sun, Yuqian Feng, Kaibo Guo, Ying Zhu, Dawei Huang, Shanming Ruan

**Affiliations:** 1 The First Clinical Medical College of Zhejiang Chinese Medical University, Hangzhou 310053, Z.J. China.; 2 Department of Medical Oncology, The First Affiliated Hospital of Zhejiang Chinese Medical University, Hangzhou 310006, Z.J. China.; 3 Department of Traditional Chinese Medicine, The First Affiliated Hospital of Zhejiang Chinese Medical University, Hangzhou 310006, Z.J. China.

**Keywords:** Colorectal cancer, gut microbiota, Zheng-Qi-Kui-Xu syndrome, Xie-Du-Yong-Sheng syndrome, Traditional Chinese medicine

## Abstract

Colorectal cancer (CRC) is considered to be closely associated with alteration of intestinal microorganisms. The purpose of present study was to investigate the distribution of gut microbiota in the distinction of microbiota dysbiosis between two disease syndromes called Zheng-Qi-Kui-Xu(ZQKX) and Xie-Du-Yong-Sheng (XDYS). First, From February 2019 to June 2019, CRC patients presenting to the oncology department of Zhejiang Province Hospital of TCM who met the established inclusion and exclusion criteria were enrolled in this prospective study. After fresh stool specimens of healthy volunteers and CRC patients with ZQKX or XDYS syndorme were collected, 16S rRNA gene amplification and sequencing could be used to identify the diversity and abundance of gut microbiota among groups. The results demonstrated that the composition of the microbiota in general control group was superior to those in experimental groups. At the phylum level, a significantly increased abundance of Bacteroides was observed in healthy volunteers. At the class level, Erysipelothrix decreased while Lactobacillaceae showed increased abundance in the ZQKX group compared to healthy controls. At the family level, Prevotella Shan and Collins decreased while Streptococcus significantly increased in patients with XDYS syndrome compared to healthy subjects. Five differential taxa were identified between ZQKX and XDYS syndromes. We suggest that the gut microbiota contributes to the distinction between the two TCM syndromes of CRC, which can be used as a biological basis of TCM syndrome differentiation treatment in CRC.

## Introduction

Colorectal cancer (CRC) has the highest incidence of digestive system cancer in the world 1. As an integral part of comprehensive treatment in CRC, Traditional Chinese medicine (TCM) has a significant effect in alleviating the side effects of radiotherapy and chemotherapy, such as improving the quality of patients' life, preventing tumor recurrence and metastasis and so on. Huang et al. 2 found that in the meta-analysis consisted of twenty-eight studies, the Traditional Chinese Medicine named Kangai Injection, could increase clinical effectiveness, improve quality of life, alleviate adverse reactions, and prolong survival time in advanced CRC patients receiving chemotherapy, which suggested combining Western medicine with TCM in a collaboratively positive way could be effective to fight tumors. Syndrome differentiation and treatment is the basic principle of TCM diagnosis. However, the clinical diagnosis of CRC has not yet established a unified system, which lacks of objective material indicators as the basis, and the existence of subjective, empirical issues, so the biological basis of TCM diagnosis and treatment in CRC is imperative.

The gut microbiota participates in various physiological activities, such as digesting and absorbing nutrients, collecting energy, metabolizing dietary component, regulating immunity and processing host or microorganism-derived chemicals 3. With the in-depth exploration in the physiopathological mechanism of CRC, gut microbiota disorder has attracted more attention as one of its pathogenesis mechanisms. Sinha et al.^4^ demonstrated that feces from CRC cases related to microbial metabolites strongly, and Flemer et al.^5^ found that CRC patients' microbial structure between the distal and proximal large intestine was significantly different from that in control group as well. Also, studies showed that Chinese medicine and gut microbiota can interact in the human body and thus affect the occurrence and development of diseases 6.

**Figure 1:**
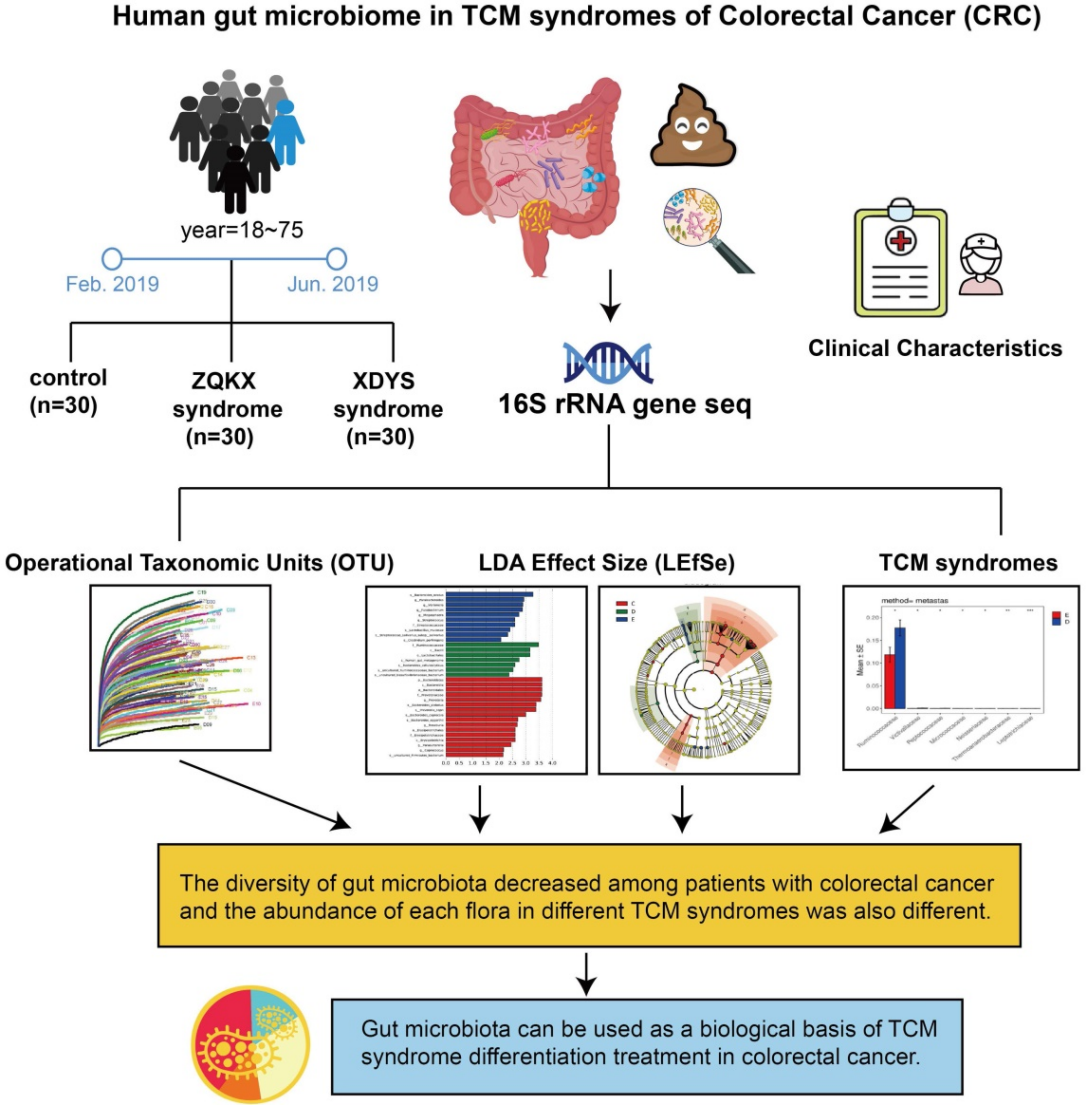
Human gut microbiome in TCM syndromes of Colorectal cancer (CRC).

Researchers suggested that CRC has a long course in disease. As the evil qi (cancer) continues to invade, the positive qi in human body is eroded. Therefore, the TCM syndrome evolved from the large intestine cancer with syndrome of internal binding of static blood and poison to spleen and kidney qi depletion. According to the stool samples of patients with Zheng-Qi-Kui-Xu syndrome (syndrome of positive qi deficiency, ZQKX) and Xie-Du-Yong-Sheng syndrome (syndrome of static blood and poison exuberance internally, XDYS), the study intends to exhibit the relationship between the syndrome classification and gut microbiota for formulating a viable biological basis of TCM diagnosis and treatment in CRC. The design is shown in Figure 1.

## Materials and methods

### Patient recruitment

All cases and specimens were collected from February 2019 to June 2019 in the oncology department of the Zhejiang Province Hospital of TCM and all patients were informed of the contents of the experiment and signed the informed consent. We promise that the study was performed according to the international, national and institutional rules considering clinical studies. The study protocol was approved by the Ethics Committee of the Zhejiang Province Hospital of TCM (2020-KL-050-01).

Inclusion criteria for the general control group included the following: (a) without using probiotics or antibiotics in the 4 weeks prior to taking stool samples; (b) being 18-75 years old; (c) having no nausea, vomiting, diarrhea and other gastrointestinal discomfort within the previous month. The exclusion criteria for the experimental groups included the following: (a) below the age of 18 or above the age of 75 years; (b)pregnant or lactating women; (c) use of probiotics or antibiotics in the 4 weeks prior to taking stool samples; (d) patients whose feces are discharged through the fistula; (e) other autoimmune diseases; (f) uncooperativeness.

### Diagnostic criteria

All patients were confirmed by operation and pathology and had no history of other malignancies besides a CRC diagnosis. The classification and grouping of cases were carried out according to Guideline for Diagnosis and Treatment of Tumor in TCM by CACM (China Association of Chinese Medicine) 7. The identification of syndromes must be present with the main symptoms and two or more accompanying symptoms. 1 point for an accompanying symptom and 2 points for tongue coating or pulse condition. We can distinguish these two syndromes according to patients' clinical manifestations, tongue coating and pulse condition (Table 1).

### DNA samples test

The stool samples collected from volunteers were store at -20°V, and the 200 mg stool samples were weighed to put into the 2-ml centrifuge tube. The extraction of DNA used the QIAamp DNA stool mini kit produced by the German Qiagen company, and the specific steps were conducted according to the directions. After adding ASL, incubate in a 95°C water bath for 5 min to increase the efficiency of Gram-positive bacteria in the feces. The final extracted DNA was dissolved in 50μL AE. The extracted total DNA was tested for concentration, purity, and integrity by 1% agarose gel electrophoresis and NanoDrop2000 ultramicro spectrophotometer, and then saved at 4°C for backup.

### PCR amplification

The primer sequences were designed into 357F (ACTCCTACGGRAGGCAGCAG) and 806R (GGACTACHVGGGTWTCTAAT). To improve the accuracy and reliability of the following analysis, ten samples were selected with a random method to produce the lowest cycle number in the pre-experiment, while most samples could be expanded to suitable concentration products. After that, the TransGen AP221-02, TransStart Fastpfu DNA Polymerase, 20-μl reaction system was used in the formal experiment. According to the quantitative results of the preliminary sample detection electrophoresis, the PCR products were quantitatively detected with a micro fluorometer, and then mixed based on the sequencing volume of a single sample.

### Bioinformatics analysis

#### Majorizing sequence

Paired-end DNA were sequenced through Illumina Miseq, and the sequencing results were transformed into images, which were extracted according to the barcode tag complete matching method. After that, Trimmomatic, Fash and Mothur software were applied for processing, splicing and optimizing sequences respectively.

#### OTU analysis

Operational taxonomic unit (OTU) is an operational definition set for classifying a certain taxonomic unit (omain, kingdom, phylum, class, order, family, genus, species) in order to facilitate analysis in phylogenetic or population genetics research. OTU clustering was performed on the nonrepetitive sequence (without the single sequence), and the chimera was eliminated during the process to obtain the optimal sequence of the OTU subsequently, which was selected in the representative sequences according to 97% similarity.

#### Taxonomic analysis

Based on taxonomic information, statistical analysis of the community structure was conducted at the classification level of phylum, class, order, family, genus, species.

#### Rarefaction curve

The majorizing sequence was randomly selected in the sequence of the OTU at the level of 97% similarity and the rarefaction curve was constructed by the corresponding OTU number and the selected sequence number. The diagram was made by the R language tool.

#### Alpha diversity analysis

The Chao1 algorithm in mothur software was performed to estimate the number of species by calculating the index of OTU in the sample.

### Community histogram

The composition of community structure at different classification levels was obtained by the results of taxonomic analysis, and diagrams were made by the R language tool.

### Multi-level species differences analysis

Linear discriminant analysis effect size (LEfSe) analysis software was performed to linear discriminant analysis on samples with different grouping conditions according to the taxonomic composition. Finally, LDA was used to screen the communities or species that have significant impacts of differences in the sample division.

### Difference analysis between groups

Samples were analyzed and compared among the general control group and experimental groups at each level in metastat software to find out the different bacterial types with a certain level of difference in the abundance of flora. The default threshold was 0.05.

### Statistical analysis

Statistical software (SPSS 22.0) was used to analyze and process the data to calculate the difference of the flora data among the general control group, the ZQKX group and the XDYS group. The metering data are represented by the average±standard deviation (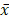
±s). The comparison between two independent samples is analyzed by t test and the differences among groups were analyzed by Wilcoxon rank-sum test. Differences were considered statistically significant (*p<0.05*), significant (*p<0.01*) and not statistically significant (*p>0.05*).

## Results

### General information

A total of 90 cases were collected in this study, including 30 cases ofin the general control group, 30 cases in the ZQKX group and 30 cases in the XDYS group. There were 47 males and 43 females, all ranging in age from 17 to 75 years, with an average of 56 years old (55.92±10.70). All volunteers provided informed consent before participating in the experiment. Among all the individuals, there was no significant difference in any clinical factor such as gender, age, or inflammation location (*p>0.05*) (Table 2).

### DNA test and identification of PCR amplification

A total of 90 samples were sequenced in this study. We tested the concentration, purity and integrity of the extracted DNA, and the quantitative and quality control of fecal DNA samples indicated that proper DNA integrality. The secondary PCR amplification product had a single band with moderate brightness, which could be used for subsequent gel recovery experiments and qPCR quantification.

### Serial data statistics

A total of 90 cases were collected in this study, including 30 cases in the general control group, 30 cases in the ZQKX group and 30 cases in the XDYS group. Based on the general principles of systems genetics and population genetics, we received 2427630 optimization sequences accurately after removing sequences containing repeated and fuzzy bases that affected the quality of analysis.

### Sequencing depth and the analysis of sample size

**Figure 2:**
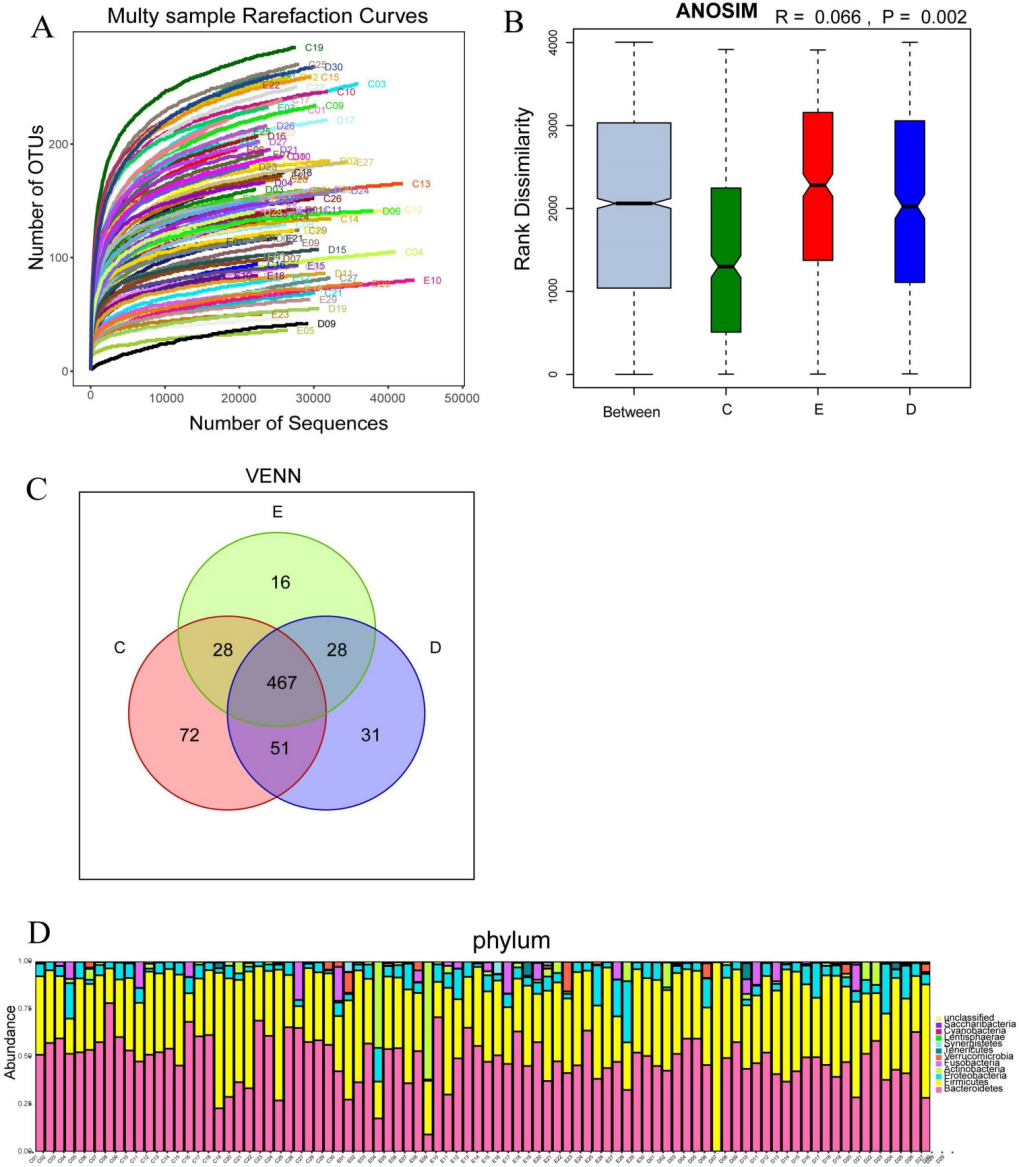
Multy sample rarefaction curves for comparing the abundance of diverse species (A); Boxplot of similarity analysis for identifying the existence of differences between groups (B); Venn diagram for indicating the common and specific characteristics among three groups (C); Histogram of the abundance distribution of species at the level of phylum (D).

The number of OTUs clustering in 90 samples of this study was 714, including 199 species, 138 genus, 47 family, 30 order, 18 class, 11 phylum, 1 domain. With the curve flattening out, more data had a marginal contribution to the discovery of new OUTs, which suggested that the study could be performed with reasonable sample collection and high species richness (Figure 2A). In addition, the similarity analysis showed that the difference between the groups was greater than the difference within the groups (R=0.066, *p<0.01*), which meant the amount of data in this study was reasonable to objectively reflect the vast majority of microbial information in the intestinal tract of each group (Figure 2B). Venn diagram (VENN) indicated the number of OTUs which were common and unique to each research group. The total number of OTUs in this study was 469, in which the OTU's number of the general control group was significantly higher than those of the experimental groups (72 vs 31,16) (Figure 2C). The abundance diversity histogram of the top ten species at the door level basing on the observation of the sample community's structure suggested that the proportions of Bacteroidetes, Firmicutes, Proteobacteria and Actinobacteria which were the predominant flora in most samples were different in each group (Figure 2D).

### Diversity analysis

We performed the Wilcoxon rank-sum test for the Chao index of the three groups, which showed that the bacterial diversity of the stool samples of the general controls was the richest, while the XDYS group was poorest. The difference among the general control group, the ZQKX group and the XDYS group were statistically significant (*p<0.01*) (Figure 2).

### Analysis of species' differences between groups

#### Discriminant analysis of community differences among LEfSe

**Figure 4:**
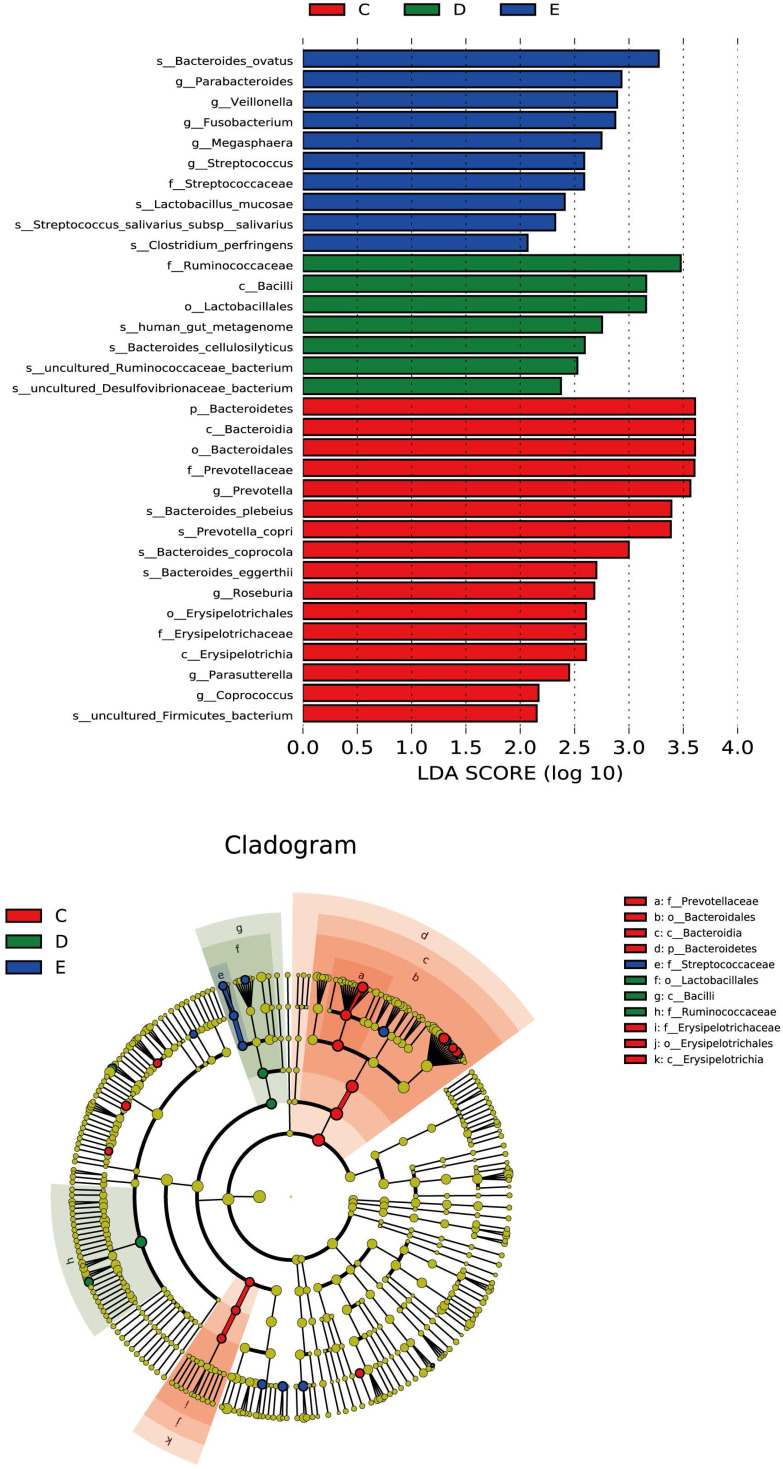
Histogram and cladogram of Linear discriminant analysis effect size (LEfSe) analysis among the general control group and the experimental groups.

From the inside to the outside of the concentric circle are phylum, class, order and family, the nodes of which correspond to the specific flora of the different groups. From the LEfSe diagram (Figure 4), it was found that there was specificity for the following in each group and level: Bacteroidetes in the general control group on the phylum level; Erysipelotrichia in the general control group; Bacilli in the ZQKX group on the class level; Lactobacillales in the ZQKX group on the order level; Prevotellaceae in the general control group; Ruminococcaceae in the ZQKX group; Streptococcaceae in the XDYS group on the family level.

#### Nonparametric test based on species information

**Figure 5:**
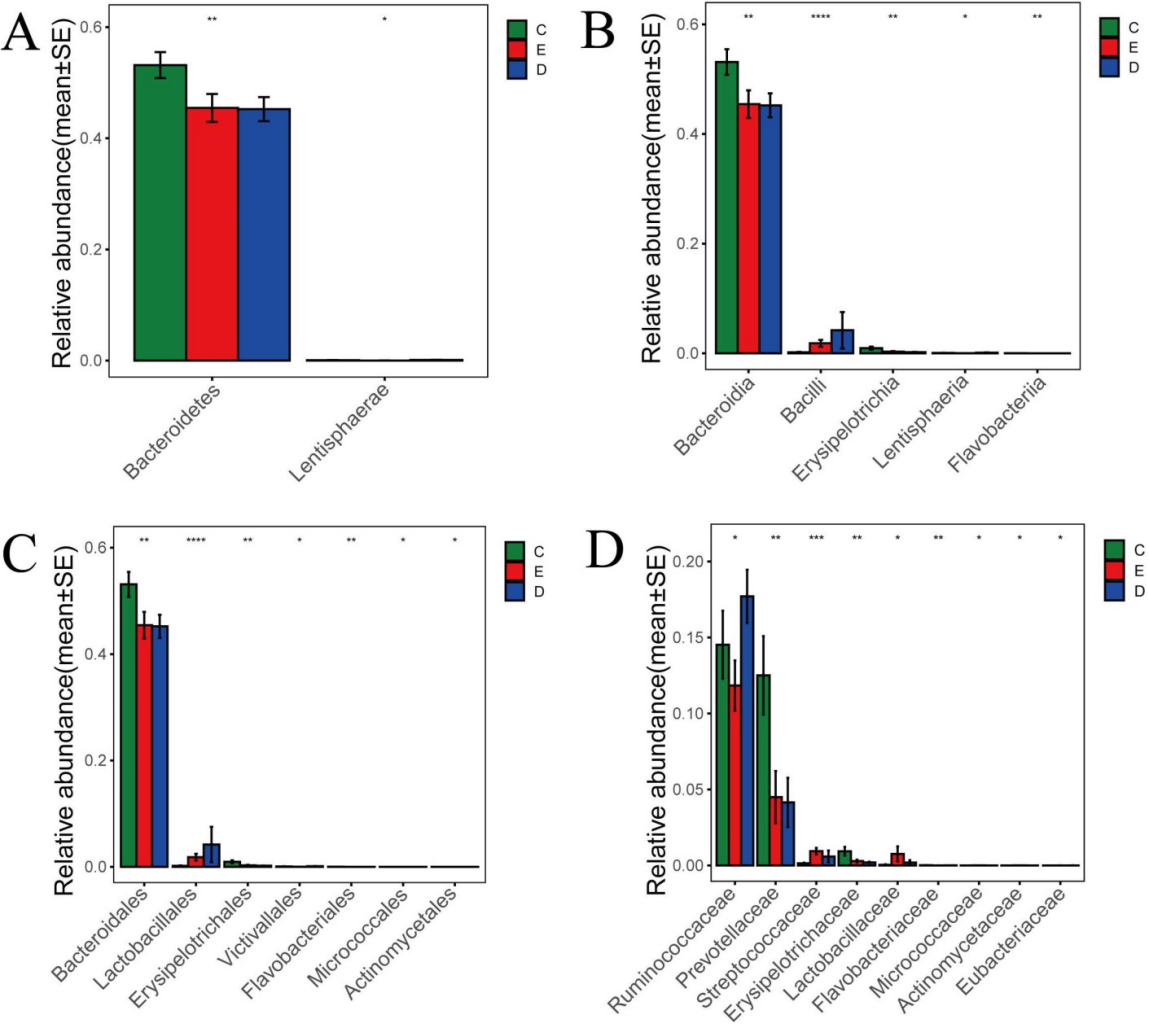
Histograms of differences in the abundance of species analyzed by Wilcoxon rank-sum test among three groups at the levels of phylum (A), class (B), order (C) and family (D).

According to the histogram of phylum (Figure 5A), we found that Bacteroidetes accounted for 45.2% and 45.5% of the total amount of bacteria, respectively, in the ZQKX and the XDYS group, which was lower than that in the general control group, which accounted for 53.2% (*p*<0.01). The abundance of Lentisphaerae in the ZQKX group was higher than that in the other two groups (0.12% VS 0.07%, 0.02%) (*p*<0.05). These differences were both statistically significant. But the comparison of difference on Lentisphaerae between two groups was unconspicuous in statistics.

According to the histograms of class and order (Figure 5B and 5C), we found that Erysipelotrichales accounted for 0.2% and 0.3% of the total amount of bacteria, respectively, in the ZQKX and the XDYS group, which was lower than that in the general control group, which accounted for 0.9% (*p*<0.01). Lactobacillales, belonging to Bacilli, accounted for 4.2% of the total amount of bacteria in the ZQKX group, which was higher than in the general control group (0.2%) and in the XDYS group (1.8%) (*p*<0.01). The abundance of Actinomycetales in the XDYS group was higher than that in the other two groups (0.008% VS 0.005%, 0.002%) *(p*<0.05). These differences were all statistically significant. However, the comparison of difference on Victivallales, Flavobacteriales and Micrococcales between groups were not noticeable in statistics.

According to the histogram of family (Figure 5D), we found that Prevotellaceae accounted for 4.2% and 4.5% of the total amount of bacteria, respectively, in the ZQKX and the XDYS group, which was lower than that in the general control group, which accounted for 12.5% (*p<0.01*). Streptococcaceae accounted for 0.95% of the total amount of bacteria in the XDYS group, which was higher than in the general control group (0.14%) and in the ZQKX group (0.59%) (*p*<0.01). The abundance of Ruminococcaceae in the ZQKX group was higher than that in the other two groups (17.7% VS 14.5%, 11.8%) (*p*<0.05). These differences were all statistically significant. But the comparison of difference on Eubacteriaceae between groups were not significant in statistics.

### Comparison of Metastats differences between TCM Syndrome

**Figure 6:**
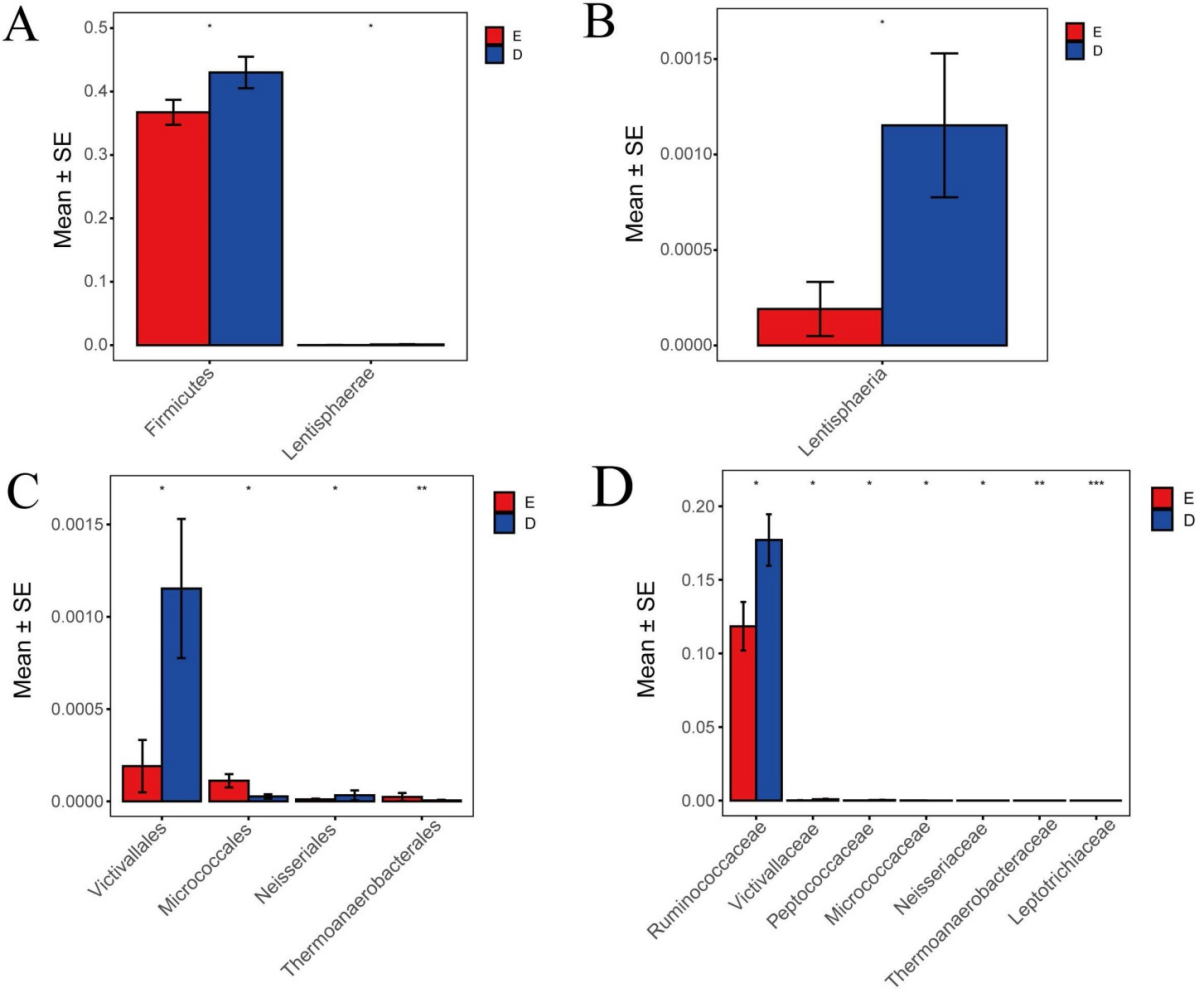
Histograms of differences in the distribution of species between the ZQKX group and the XDYS group performed by Metastats software at the levels of phylum (A), class (B), order (C) and family (D).

In this study, Metastats indicated species differences (phylum, class, order, family) with comparison and analysis between ZQKX group and the XDYS group. According to the histograms of phylum and class (Figure 6A and 6B), the differences of Firmicutes and Lentisphaerae were both statistically significant (43.0%>36.7%, 0.12%>0.02%) (*p*<0.05). According to the histograms of order and family (Figure 6C and 6D), the differences of Thermoanaerobacteraceae and Leptotrichiaceae were both statistically significant (0.0001%<0.0021%, 0.0005%<0.0024%) (*p*<0.01). However, the statistical differences in Micrococcaceae, Ruminococcaceae, Victivallaceae, Neisseriaceae and Peptococcaceae between the experimental groups were not evident.

## Discussion

### Research status of the relationship between CRC and gut microbiota

Gut microbiota is symbiotic with the human gastrointestinal tract, participating in various physiological processes in host's nutrition absorption and metabolism 8, which plays an important role in maintaining the intestinal homeostasis. Under normal conditions, the gut microbiota coexisting with the host remains in a dynamic balance, while the broken may result in certain diseases happening 9. Peters et al. 10 found that the fecal species' abundance in patients with CRC was evidently lower than that in healthy people (*p*=0.03), suggesting potential dysbiosis. Sunny et al. 11 found that the proportions in the experimental group of Ki-67 positive cell (*p*<0.05) and the large intestine developed into atypical hyperplasia (*p*<0.05) or macroscopic polyps (*p*<0.01) increased dramatically compared with the control by feeding the CRC patients' feces to the mice. Above all, gut microbiota is closely related to the occurrence and development of CRC.

With the progression of clorectal cancer, the pattern evolves from XDYS syndrome to ZQKX syndrome. The former syndrome is caused by the accumulation of various pathological products such as damp heat, blood stasis, phlegm and dampness and the latter one is involved in the dysfunction of water-liquid metabolism system and the consumption of positive qi, showing the weakness in spleen, liver and kidney. ZQKX syndrome is a deficiency syndrome, while the XDYS syndrome is an excess syndrome. The similarity analysis proved that there were distinctions between the typical types of CRC selected as the research objects, so the gut microbiota connects with the syndrome distribution in TCM, used as a biological basis of TCM syndrome differentiation treatment in CRC.

### Characteristics of bacterial flora in patients with CRC and the healthy population

Gut microbiota's diversity and richness are beneficial to maintain the stability of the micro-ecosystem as well as enhance the resistance to bacteria. Many studies showed that the diversity of gut microbiota decreased among patients with CRC 12. The Chao index revealed that the diversity of microbial communities in the general control group was higher than those in ZQKX and XDYS groups. The healthy human body relies on the abundant micro-ecosystem so as to maintain the necessary nutrients and metabolism, and strengthen various metabolic and immune activities between the human body and the outside world. In the early stage, heat, poison, dampness, stasis and other evil poisons were infused into the gastrointestinal tract and infiltrated the intestines with the fights between positive qi and evil qi, which severely blocked the qi-blood conduction and the large intestine metabolism and accumulated pathological products, resulting in the microbial environment destroyed, the number of beneficial bacteria reducing and the tribe diversity significantly affected. Later, the condition-causing pathogens multiplied with the disease course increasing, so the gut microbiota diversity in ZQKX group was higher than that in XDYS group and this result demonstrated that the predominant phylums were Bacteroidetes, Firmicutes, Proteobacteria and Actinobacteria, which was similar to many experimental results at home and abroad. Among them, Bacteroidetes and Firmicutes accounted for more than 85% in the total amount of gut microbiota, which belonged to the dominant phylums absolutely 13. Everard A et al. 14 suggested that these two bacteria might be involved in carbohydrate metabolism and energy absorption processes and the ratio ranges were related to the formation of human bowel movements, fecal traits and obesity. Comparing healthy volunteers with patients in CRC, Prevotella and Erysipelothrix were relatively abundant in addition to the Bacteroidetes. The abundance of Prevotella in the general control group was significantly higher than those in experimental groups (12.5% vs 4.2%, 4.5%). Prevotella has probiotic properties, existing on the mucosal surface where the human body communicates with the outside world such as oral mucosa, upper respiratory tract mucosa, intestinal mucosa, and reproductive tract mucosa. Prevotella helped the intestines abstract from plant-based diets furthest, so the high expression of Prevotella reflected that normal people might intake more fiber and other plant compounds 15.

### Analysis of bacterial characteristics in XDYS group

The predominant bacteria in XDYS group were Streptococcus, Leptotrichia and Micrococcus. Although the abundances of Leptotrichia and Micrococcus were not high in XDYS group (0.0021%, 0.01%), there were statistical distinctions between the general control group and the ZQKX group. Leptotrichiaceae belongs to the Fusobacteria. Kostic et al. 16 observed a strong correlation between the abundance of Fusobacteria and the proinflammatory markers' expression like COX-2, suggesting that Fusobacterium could create a pro-inflammatory microenvironment conducive to CRC through recruiting tumor-infiltrating immune cells. Micrococcales belongs to the Actinobacteria, some of which were recognized as opportunistic pathogens associated with colon-related diseases such as colon cancer and Crohn's disease 17. As a pyogenic strain, Streptococcus had an advantage in XDYS syndrome. Siyang et al. 18 found that the abundance of Streptococcus in cancer tissues was higher than that in adjacent non-cancerous ones and Streptococcus could aggravate the tumor microenvironment and accelerate the development of CRC with inflammatory factors such as cyclooxygenase-2 (COX-2), interleukin-1, and interleukin-8. The Wnt/β-catenin signaling pathway could regulate cell proliferation, polarity, embryonic development and tissue homeostasis, and its abnormal activation contributed to various diseases including malignant tumors 19. Ren et al.^20^ found that Streptococcus metabolites had the ability to enhance DNA replication and promote colon cancer cell proliferation and migration in the methods of activating the Wnt signaling pathway and facilitating the downstream tumor-related target gene c-myc mRNA to overexpress. Schwabe et al. 21 reported that gut microbiota was the main driver to cause colon inflammation and the inflammatory environment was directly related to CRC's come up. Therefore, the abundance of Streptococcus, Leptotrichia, Micrococcus and other bacterial groups increasedly facilitated the inflammatory microenvironment formation and provided conditions for the occurrence and metastasis of cancer cells in XDYS group.

### Analysis of bacterial characteristics in ZQKX group

The Firmicutes has been confirmed to be the dominant flora in the patients' intestine with CRC, whose abundance in ZQKX group was obviously higher than that in XDYS group. The circumstance that patients with deficiency syndrome had a longer course of disease and a relatively more serious condition, resulted in the larger proportion of this phylum. In addition, the Neisseriaceae, Lentisphaeraceae, Victivallaceae, Ruminococcaceae, and Peptococcaceae were also luxuriant in ZQKX group. The abundance of Lactobacillales was considered as an individual difference due to only one sample in which there was the distinction within the ZQKX group. Neisseriaceae belongs to the class β-Proteobacteria and it has been demonstrated to multiply coupled with the microbiological imbalance during disturbances in human intestinal metabolism. Studies reported that the degree of metabolic disorders in the gut microbiota of Bangladeshi children was closely related to the abundance of Proteobacteria and the researcher observed that the dominant bacteria were Proteobacteria in the children's intestines with malnutrition from time to time 22.

As for differences between groups about the abundance of Erysipelotrichaceae, Ruminococcaceae, Thermoanaerobacteraceae, Victivallaceae, Peptococcaceae and Leptotrichiaceae, there are few relevant studies presented and some experimental results even are completely opposite in a few studies, suggesting little references to supply its effects or pathogenic mechanism on human intestinal function. Therefore, more sample sizes and experimental studies are still in need to clarify the specific significance of gut microbiota in the occurrence and development of CRC and to take a connection between the TCM syndrome classification and gut microbiota for offering new methods in the motherland's medical syndrome differentiation and treatment system.

## Conclusion

Our study suggested that the diversity of gut microbiota decreased among patients with CRC and the abundance of each flora in experimental groups was also different. The predominant bacteria in ZQKX group were Neisseriaceae, Lentisphaeraceae, Victivallaceae, Ruminococcaceae, and Peptococcaceae. While in XDYS group, Streptococcus, Leptotrichia and Micrococcus have been confirmed to be the dominant bacteria. So the gut microbiota contributes to the distinction between the two TCM syndromes of CRC, which can be used as a biological basis of TCM syndrome differentiation treatment in CRC.

## Figures and Tables

**Figure 3:**
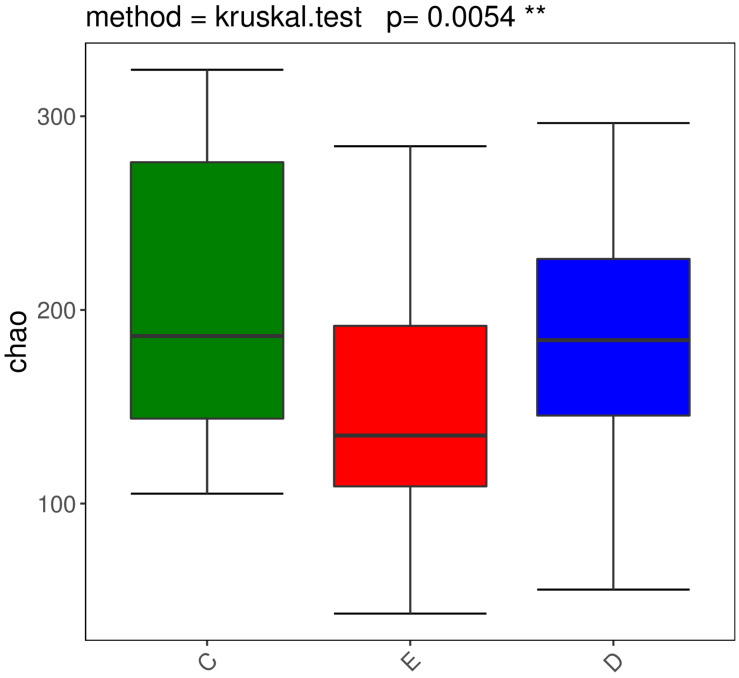
Boxplot of the Wilcoxon rank-sum test for the Chao index.

**Table 1:** The classification of ZQKX syndrome and XDYS syndrome

	ZQKX Syndrome	XDYS Syndrome
Main symptoms	Intestinal irritation symptoms and changes in bowel habits, blood in the stool, intestinal obstruction, abdominal mass and anemia, weight loss, fever, fatigue, etc.	
Accompanying symptoms	Loose stools with blood; prolapse of the anus; loss of appetite; dizziness and paleness; fatigue.	A burning sensation around the anus; bloody stools with white mucus; tenesmus; abdominal pain; loss of appetite; thirst.
The tongue coating	Pale and thin	Greasy and slightly yellow
The pulse	Weak and thready	Slippery and rapid

**Table 2:** Characteristics of healthy volunteers and CRC patients

	General control group (n)	CRC group	
		ZQKX group (n)	XDYS group (n)
**Cases**	30	30	30
Sex ratio (F/M)	19/11	15/15	9/21
**Duration of disease (years)**			
<2		14	13
2-4		9	13
>4		7	4
**Inflammation location**			
Rectum		13	16
Left colon		7	1
Whole colon		10	13
Smoking history (Y/N/unclear)		10/20/0	12/18/0
Drinking history (Y/N/unclear)		9/11/0	7/23/0
